# Agroforestry protects arable crops from climate shock during critical early-season phenological stages

**DOI:** 10.1007/s13593-026-01129-3

**Published:** 2026-07-23

**Authors:** Colin R. Tosh, Christian Gossell, Isabelle Lecomte, Marie Gosme, Jonathan M. Eden, Christina den Hond-Vaccaro, Will Simonson, Felix Herzog, Christian Dupraz

**Affiliations:** 1 Organic Research Centre, ADAS, Spring Lodge, 172 Chester Road, Helsby, Cheshire, WA6 0AR UK; 2 Centre for Agroecology, Water and Resilience, Coventry University, Ryton Gardens, Wolston Lane, Ryton-on-Dunsmore, Coventry, CV8 3LG UK; 3 ABSys, Univ Montpellier, Cirad, INRAE, Institut Agro Montpellier, Montpellier, France; 4 Agricultural Landscapes and Biodiversity, Agroscope, Schwarzenburgstrasse 161, Bern, 3003 Switzerland

**Keywords:** Agroforestry, Climate change adaptation, Crop resilience, Simulation modelling, Land equivalent ratio (LER)

## Abstract

Climate change poses significant threats to European agricultural production, with increasing frequency and severity of adverse weather events impacting crop yields. Agroforestry, the integration of woody elements into agricultural systems, is recognized as a vital agroecological approach for both climate change mitigation and adaptation*,* yet key mechanistic and long-term performance questions remain unresolved. This study utilized the mechanistic Hi-sAFe model to simulate 100 years (2001–2100) of silvoarable agroforestry performance at Wakelyns farm in southeastern England, focusing on winter wheat (*Triticum aestivum*) and pea (*Pisum sativum*) yields under intermediate (Representative Concentration Pathway 4.5) and very high (Representative Concentration Pathway 8.5) emissions scenarios. The research assessed yield stability, underlying microclimatic and phenological mechanisms, and long-term land-use efficiency (land equivalent ratio). This study demonstrates for the first time that agroforestry functions as a climate shock absorber by protecting crops during a critical early-season phenological window, preventing catastrophic yield failures under climate change scenarios. These protective effects were mechanistically linked to microclimatic modification, likely shade, provided by newly emerged walnut (*Juglans regia*) leaves during the early stages of crop flowering and grain filling, rather than during peak summer heat. While overall yield stability assessed statistically was not significantly enhanced, the mitigation of extreme downside risk represents a profound benefit for farm resilience. Analysis of land equivalent ratio revealed a substantial initial productivity lag, with consistent land equivalent ratio > 1 achieved only after 80 years for wheat and 40 years for pea, highlighting economic adoption barriers but also the potential for optimized system design and adaptive management to accelerate productivity gains. Overall, these results identify a previously unreported mechanistic and temporal basis for agroforestry’s capacity to buffer temperate arable crops against climate-induced yield shocks.

## Introduction

### Challenges of climate change for European agricultural production

Climate change threatens global food production in the medium to long term (Kummu et al. 2021). European agriculture will also be affected, with regional variability in climate impacts (Orlandini and Nejedlik 2012). A northward shift of agro-climate zones in Europe is already observed and expected to accelerate further (Ceglar et al. 2019). In Northern Europe, climate change may result in yield increase due to generally warmer temperatures and longer vegetation periods, in particular for C3 crops (Hristov et al. 2020; Faye et al. 2023). The increased CO_2_ levels in the atmosphere may also enhance photosynthesis, which may result in crop yield increases if there are no other stressors (Ainsworth and Long 2021; Rezaei et al. 2023). Farmers across Europe have started to adapt their agricultural practices to meet climate impacts, particularly by altering timing of cultivation and crop/cultivar selection (Olesen et al. 2011) and by experimenting with new crops, such as paddy rice (*Oryza sativa*), olive (*Olea europaea*), and almond (*Prunus dulcis*) trees (Adrian Reutimann et al. 2020; Widmer et al. 2025).

Yet, overall, temperature and precipitation trends since 1989 have already reduced wheat and barley (*Hordeum vulgare*) yields in Europe by 2.5% and 3.8% in 2009 and yields will reduce further with continued warming (Moore and Lobell 2015; IPCC 2022). A European quantitative biophysical, agro-economic modelling analysis expects European grain maize (*Zea mays*) yields to decline between 1 and 22% and wheat yields in Southern Europe to decrease by up to 49% by 2050 under the very high greenhouse gas emissions scenario defined by Representative Concentration Pathway (RCP) of 8.5 W/m2 (Hristov et al. 2020).

Adverse weather events are expected to become more frequent, rendering adaptation strategies difficult. For instance, an agro-climate modelling study projects adverse weather events at least once every 2 years for one-half of the arable land area of Europe by 2090 under RCP8.5 (Trnka et al. 2015)—rendering adaptation strategies difficult. The severity of heatwave and drought impacts on crop production roughly tripled over the last 50 years, from −2.2% (1964–1990) to −7.3% (1991–2015) (Brás et al. 2021). A meta-study on crop yields in Germany showed that drought is the most important climate extreme in German crop production and has become more relevant in the recent past (Schmitt et al. 2022). Apart from drought and heatwaves, heavy precipitation events, cold waves, and hail are also relevant weather extremes (Brás et al. 2021; Hulton and Schultz 2024). On top of direct adverse impacts, climate change also indirectly affects crop production, for instance, through soil degradation. Soil loss by water erosion is projected to increase by 13–22.5% in the EU and UK by 2050 (Panagos et al. 2021).

### Agroforestry as part of climate change mitigation and adaption

Agriculture contributes to and is affected by climate change. Applying agroecological principles mitigates greenhouse gas emissions and potentially accomplishes adaptation to climate change impacts on crop production (Dittmer et al. 2023). Agroforestry, the integration of woody elements in agriculture, is an agroecological concept and practice regarded as being both an approach to mitigate and adapt to climate change impacts (Gupta et al. 2023). As for mitigation, Kay et al. (2019) estimated that if 10% of European farmland were converted to agroforestry, up to 43% of the agricultural sector’s greenhouse gas emissions could be compensated. Regarding the adaptation to climate change, it has been proposed that trees and shrubs exert “buffer functions,” allowing agroforestry systems to persist in fragile environments (van Noordwijk et al. 2021). These buffer functions include soil protection and the creation of a beneficial microclimate, where the temperature range of microclimatic effects of trees exceeds localized temperature increases associated with a warmer world (van Noordwijk et al. 2021). With their deep roots, trees are able to both store and access more water in the soil than other vegetation, and forested regions exhibit more intense moisture recycling than non-forested regions (Ellison et al. 2019). Empirical evidence for these buffering capacities of agroforestry stems mainly from tropical regions, where harsher environmental conditions have triggered such research (Miller et al. 2020). In temperate regions, climate extremes have only recently been recognized as a threat to agricultural production, and this risk will increase in the future. Empirical evidence of beneficial effects of trees in moderating those extremes is scarce (but see Nasielski et al. 2015; Blanchet et al. 2022; Vaccaro et al. 2022; Bachakdjian et al. 2024; Koch et al. 2025) and in situ experiments will only yield results after years or decades, due to slower tree growth than in the Tropics. In contrast, expert interviews and virtual experiments using modelling approaches can yield information about the potential protective influence of trees on crops in agroforestry systems much sooner.

Two notable studies have used biophysical agroforestry simulation models to study the capacity of agroforestry to buffer arable crops under climate change in more northerly latitudes. Giannitsopoulos et al. (2025) calibrated the EcoYield-SAFE model (Burgess et al. 2025) and used it to project grass, arable, woodland, and agroforestry outcomes under RCP4.5 and RCP8.5 for 2020–2060 and 2060–2100 in Northern Ireland. They found that elevated CO₂ broadly offset the negative impacts of higher temperatures and drought, leading to increased grass, crop, and timber yields, and relatively stable land-equivalent ratios (LER) in silvopastoral and silvoarable systems. Pure arable and grassland systems were predicted to lose 1.02–1.18 t C ha⁻^1^ year⁻^1^ and 0.43–0.55 t C ha⁻^1^ year⁻^1^, respectively, whereas agroforestry and woodland systems maintained or gained soil organic carbon over 40 years, with longer-term SOC losses reduced by tree planting. Virtual trials showed that moderate tree densities can balance understory productivity with timber volume, underscoring agroforestry’s role in sustaining yields and soil carbon under a warming, high-CO₂ future.

Secondly, a French biophysical-mechanistic model (Hi-sAFe) was applied to the growth of winter durum wheat in monoculture and in combination with hybrid walnut (*Juglans nigra* × *J. regia*) using the Intergovernmental Panel on Climate Change (IPCC) projections, representing *Past* (1951–1990), *Present* (1991–2030), and *Future* (2031–2070) climatic conditions (Reyes et al. 2021), taking into account “wet” and “dry” years with medium-sized trees. In wet years, wheat yield in agroforestry was 13% lower than in monoculture, but in dry years, wheat yield equaled that in monoculture due to reduced crop water stress, highlighting the potential of agroforestry as a climate change adaptation strategy.

While these studies demonstrate that agroforestry can buffer crop performance and sustain productivity in temperate climates, important questions remain unresolved. In particular, previous modelling has not identified the specific microclimatic or phenological mechanisms through which trees influence crop resilience, nor the timing within the crop life cycle at which these interactions exert their strongest effects. Additionally, the long-term behavior of key system metrics—such as yield stability and land-use efficiency (LER) over multi-decadal timescales—remains poorly understood. Addressing these gaps is essential for advancing a mechanistic understanding of agroforestry’s adaptive potential under future climate scenarios.

**Fig. 1:**
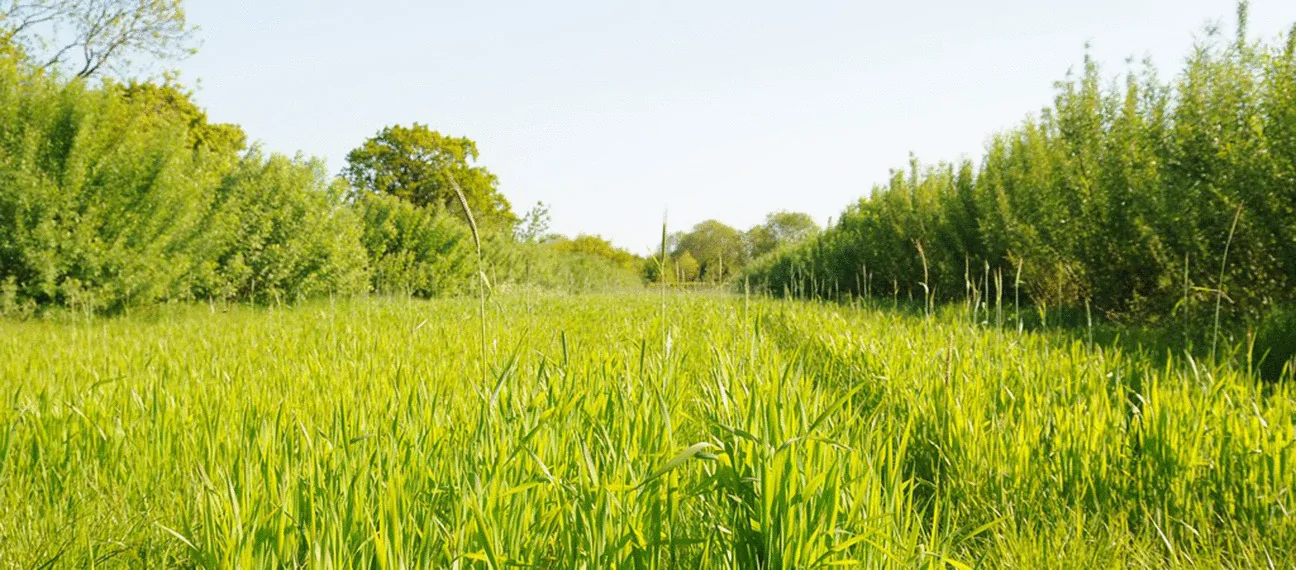
Wheat growing between willow coppice at Wakelyns Agroforestry in South-East England. Trees in such systems can protect the arable crop from environmental extremes through microclimate modification. Photocredit: Organic Research Centre.

Taking advantage of a silvoarable agroforestry pioneer farm in south-eastern England (Fig. 1), this study investigates the effects of climate change on yield stability in this region of highest importance for arable production in the UK. The following research questions were addressed:

1. Can an increase in arable crop yield stability (a component of farm resilience) be expected in agroforestry compared to open crop fields?
2. Can the biophysical mechanism underlying any pro-yield-stability effects in agroforestry be determined?
3. Is the agroforestry area modelled optimized in spatial design and management in terms of overall tree and crop productivity, i.e., is LER > 1, and how many years after tree planting does it take to achieve this milestone.

The performance of English silvoarable agroforestry over 100 years for two IPCC-defined future climate scenarios was modelled using the biophysical Hi-sAFe model (Dupraz et al. 2019). Means and variance of winter wheat and legume yield were modelled to determine if agroforestry stabilizes crop yield in the face of climate change. Advanced statistical methods were used to interrogate the model to determine which environmental variables are responsible for agroforestry’s protective effects and when in the growing season they have their effects. It is demonstrated that this process of mechanistic inference from detailed agroforestry models such as Hi-sAFe can have real-world implications for agroforestry tree choice and crop breeding.

## Methods

### Area of the site modelled

Wakelyns agroforestry is a 23-ha site in the agriculturally productive southern East Anglia region of England. Established by Professor Martin Wolfe in 1992, it is one of the oldest and most mature agroforestry sites in the UK. The site is organic and hosts a number of alley cropping areas with short-rotation willow (*Salix* spp.) and hazel (*Corylus* spp.), mixed fruit and nut trees, and mixed hardwood, and a complex arable rotation in alleys with a cross-population wheat (YQ variety) and lentil main crops and leguminous and non-legume leys (Fig. 1). It also has a stacked enterprise structure with numerous small businesses on site (bakery, kitchen, vegetable grower, willow product makers, cloth makers, and an environmental advocacy group), all using materials grown on site as principal input (https://wakelyns.co.uk/). It has generated a considerable body of farmer-facing and academic research (Smith et al. 2012a, b, 2013, 2023; Westaway et al. 2021; Smith and Westaway 2024).

**Fig. 2:**
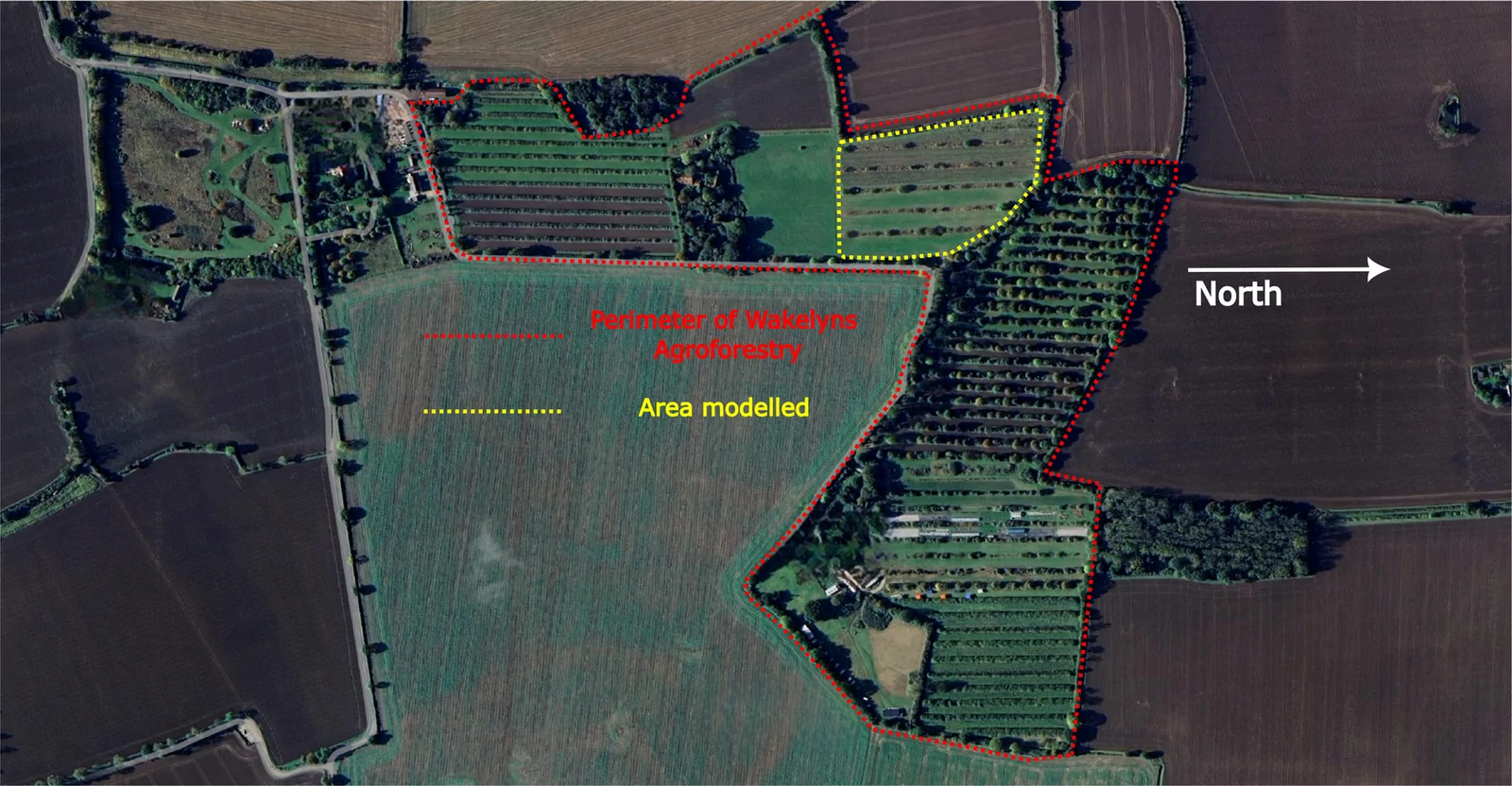
Layout of Wakelyns Agroforestry (lat 52.36048, long 1.35793) and the area of the site modelled (the 2.57ha North Field). Wakelyns Agroforestry covers 23ha. The map shown was taken from Google Maps accessed 16/03/2025. It has been subject to image manipulation to remove unwanted labels and text.

This study focuses on a silvoarable agroforestry system that was established in the North Field of the Wakelyns farm (lat 52.36162, long 1.35232, Fig. 2) in 2001. The field consists of mixed walnut and fruit plum (*Prunus domestica*) trees; however, it was modelled as a purely walnut-based system, given the current interest in this nut as a commodity crop of agroforestry systems. The arable rotation described in the “Arable rotation” section below is propagated between tree rows. Inter-tree distance within rows is 9 m and the tree understory strip is 6 m wide. Crop alleys are 15 m wide. This translated to a Hi-sAFe “scene” (Dupraz et al. 2019) of plotHeight = 9, plotWidth = 22, treeX = 10.5, treeY = 4.5, and treeCropDistance = 3, with cellWidth = 1. Tree lines are N-S aligned and the site has an elevation of 52 m. Note that crop yield was only quantified across the 1-m strip perpendicular to trees in the scene. More extensive quantification was beyond available computational resources.

### Model parameterization to local conditions

Hi-sAFe is a mechanistic, three-dimensional, process-based model that simulates dynamic interactions between trees and crops in agroforestry systems. It couples the STICS crop model (Brisson et al. 1998) per cell with a detailed tree module responsive to above- and below-ground resource heterogeneity (light, water, nitrogen), explicit root architecture, and a dynamic water table. It is driven by daily weather inputs (temperature, humidity, radiation, precipitation, wind speed), and includes management interventions and adaptive tree plasticity in canopy and root growth, enabling exploration of diverse agroforestry designs, management practices, and environmental scenarios (Dupraz et al. 2019).

The model has been applied at numerous sites across France and the rest of Europe, but the Restinclières agroforestry site in southern France has been key to its development and the majority of its applications (Mulia and Dupraz 2006; Dufour et al. 2013; Andrianarisoa et al. 2016).

Only model parameters that were modified based on field observations of the Wakelyns site are covered here. There are many other model parameters that were not measured at Wakelyns, and default values based on modelling of the Restinclières agroforestry site were used. All model parameter files for all scenarios run are available as supplementary material: Hi_sAFe_parameter_files.zip.

### Soil and water table

Accounting for local soil conditions and the water table is a prerequisite for Hi-sAFe modelling as the availability of water is a key driver of plant growth. Therefore, deep core sampling was undertaken at Wakelyns in Autumn 2022. Coring was undertaken in a small monoculture field at Wakelyns (lat 52.36020, long 1.35247) rotating between ley and cereals and unplanted with trees since the creation of Wakelyns agroforestry in the mid-1990s (Smith and Westaway 2024). It, therefore, approximated soil conditions at the establishment of Wakelyns agroforestry where land prior to first tree planting was dominated by conventional monoculture cereal propagation. A 15-cm diameter core was sunk to approximately 4 m, when dense clay precluded further penetration, and cores were removed for visual examination and sampling. Five soil layers were identified based on visual appearance, and samples were taken from each layer and sent to NRM laboratories for chemical and physical analysis (Report Number 32583-22). Key soil properties based on this soil analysis and used in the parameterization of the Hi-sAFe Wakelyns model are shown in Table SI1 of the Supplementary Information.

Another core was sunk in an agroforested area of the site and lined to form a water table sampling well. Several observations were taken prior to model parameterization but insufficient sampling was undertaken to infer seasonal trends in water table depth. Samples indicated that the water table varied around 3 m depth and a constant water table of 3.26 m depth (the average of available observations) was assumed in the model.

### Trees

Walnut trees (*Juglans regia*) planted at Wakelyns in 2001 were sampled (*n* = 17) for above-ground dimensions in Winter 2023 to derive model allometries, taking into account the actual tree management on site (Wakelyns trees are pruned every year to maintain the canopy base at 1/3 tree height). Roots are pruned at the edge of the tree line understory every 3 years and to a depth of 18 cm, and this procedure is also implemented in the model. Walnut trees were planted in 2001, and this was the start date of our simulations (day 288). Modelled trees were assumed to be 3 m high at planting with a round 2-m diameter canopy. Validation of above-ground tree growth trajectories by comparison to real tree data in 2023 is shown in Figure SI1.

### Arable rotation

The farm manager and arborist at Wakelyns were interviewed in summer 2022 regarding management practices. The following rotation is the overarching rotation that the manager indicated occurs across site, but it should be appreciated that some areas do not observe this rotation, and some alleys may be used at short notice for other purposes. Wakelyns is an organic farm and does not apply fertilizer, relying instead on nitrogen-fixing legume leys and lengthy break periods. The following account emphasizes aspects of the rotation that have been parameterized into the Hi-sAFe model to represent specifics of the Wakelyns rotation. These and additional aspects of the rotation, inherited from Restinclières, can be examined in the supplementary material.

At Wakelyns, a cross-population bread wheat called YQ (Haigh et al. 2008) is sown in October (14 October was assumed in year one of our simulations) of year one of the rotation into a tilled ley. Till depth is 18 cm and the crop is sown at 200 kg per hectare. This YQ variety is unavailable in Hi-sAFe so a winter bread wheat variety called “Talent” was used that is included in Hi-sAFe. The crop remains largely unmanaged until harvest in August of year 2. In the model, harvest was triggered by a maximum fruit water content of 0.14, which produced harvest at roughly the correct time. Straw is removed and a legume ley is immediately planted into the stubble. This is typically a mix of clover (*Trifolium *spp.), lucerne (*Medicago sativa*), birdsfoot trefoil (*Lotus corniculatus*), and sainfoin (*Onobrychis viciifolia*) but none of these species was available in the model so this ley was approximated with a monoculture of alfalfa (*Medicago sativa*) (var Luzerne). This ley remains unmanaged until April of year 5. It was assumed there would be annual seed drop and growth, and to simulate this process, a replanting of alfalfa among existing plant material was assumed in August of years 3 and 4 of the ley. In April of year 5, the ley is tilled into the soil (till depth as above) and lentil (*Lens culinaris*) is planted at 100 kg per hectare. Again, lentils are not available in Hi-sAFe so they were approximated with pea. These are unmanaged until August when they are harvested. In the model, harvest was triggered when maximum seed water content reached 0.16, which produced harvest at roughly the correct time. Hay is removed and a “weed” ley (small non-leguminous herbaceous plants) is immediately planted into the stubble. This ley was approximated with grass which self-seeded in August of each year. This ley remains unmanaged throughout years 5 and 6 of the rotation and is tilled into the soil in year 1 of the rotation for planting of winter wheat. Tree understories are largely unmanaged and dominated by grass at Wakelyns. Unmanaged grass that self-seeded each year was assumed in the model.

### Weather data and climate model scenarios

The Hi-sAFe model requires daily weather for eight variables: maximum and minimum temperature, relative humidity, evapotranspiration, precipitation, global radiation, and surface wind speed. These input variables were taken from the Regional Atmospheric Climate Model (RACMO) developed by the Koninklijk Nederlands Meteorologisch Instituut (van Meijgaard et al. 2008, 2012). RACMO has been shown to perform strongly when compared to other regional climate models (RCMs) that contribute to the European Coordinated Regional Downscaling Experiment (Jacob et al. 2014; Vautard et al. 2021). The daily input variables were made available via the CliPick climate data access tool, which is designed to facilitate the selection of climate change data for forestry and agriculture applications (Palma 2017).

RACMO data is available for historical (1951–2005) and future (2006–2100) periods. Projections for the future follow RCPs (Moss et al. 2010) that specify the concentrations of greenhouse gases that will correspond to pre-determined increases in total radiative forcing by 2100, relative to pre-industrial levels. The IPCC identifies four such pathways, RCP2.6, RCP4.5, RCP6.0, and RCP8.5, that are associated with a range of global temperature change through to the end of the twenty-first century (Van Vuuren et al. 2011). The RCPs map directly to the updated Shared Socioeconomic Pathways (SSPs) used in the most recent IPCC 6th Assessment Report (Lee et al. 2023). Here, two future climate scenarios are explored: the “intermediate” RCP4.5 scenario and the “very high” RCP8.5 emissions scenario.

Data from climate models (whether global or, as in this case, regional) are subject to inherent errors and biases. It is critical to validate model performance against real-world data and to consider whether one or more variables would benefit from a bias correction step. Output from RACMO was compared with data for corresponding variables collected on-site for the period 2013–2015: the only detailed climatic data available for Wakelyns at the time of model development. Using the observational data as a reference, quantile mapping (Cannon et al. 2015) was applied to RACMO data to correct systematic model bias. In validating the performance of quantile mapping to correct model bias, RACMO data were independently corrected following a leave-one-out cross validation approach. For most variables, the value added by the bias correction step was limited. The exception was surface wind speed, for which the bias corrected values were much more consistent with the on-site observational data. Thus, the subsequent analysis used bias corrected surface wind speed data; raw model output was taken for all other variables. Temperature data used to drive the models are included in the supplementary material: Hi_sAFe_parameter_files.zip.

### Mechanistic inference and statistics

After observing initial findings on how wheat and pea yield varies by agriculture type (agroforestry vs monoculture) and year, it was observed that some years agroforestry and monoculture produced similar arable yields and other years they produced quite different yields. The mechanisms behind such effects were examined: which environmental variables are driving yield and yield differences between agroforestry and monoculture and when in the growing season these mechanisms determine yield. This could be done by model parameter manipulation; however, this seemed potentially prohibitive in terms of computational requirements and promised to be a long and very involved process given the large number of model parameters that could be influencing yield. Instead, it was decided to take a statistical approach that takes into account the special statistical issues that can arise when analyzing model dynamics statistically and the use of complex statistical models with many interaction terms i.e., variance inflation due to multicollinearity.

The growing season of wheat was split into four periods. Period 1 was from planting until Dec 31, Period 2 was from Jan 1 to March 14, Period 3 was from March 15 to May 31, and Period 4 was June 01 to harvest. For pea the periods were: Period 1 was from planting until May 31, Period 2 was from June 1 to June 30, Period 3 was from July 1 to July 31, and Period 4 was Aug 1 to harvest. Means of daily values of precipitation, relative humidity, maximum daily temperature, and global radiation across each period were calculated to produce statistical variables: Precipitation, RelHumid, MaxDailyTemp, GlbRadiation. Additional statistical variables were Year (year of harvest) and AgType (agroforestry vs monoculture). Pea and wheat yield were the dependent variables. All two-way, three-way, and four-way interactions between Year, AgType, Period1, Period2, Period3, Period4, and the environmental variables Precipitation, RelHumid, MaxDailyTemp, and GlbRadiation were then run. The most significant terms involving the interaction between AgType*Year*Period (any single period)*Environmental variable (any one or more in interaction) combination were of primary interest. The R formula structuring this analysis is too complex to reproduce here but can be examined in the “Summary” sheet of the “Analysis” MS Excel worksheet submitted as supplementary material (starting at cell BD329).

Unsurprisingly, when generalized linear modelling (GLM) was run with this very complex statistical model with significantly correlated independent variables (confirmed by examination of the correlation matrix), significant variance inflation was observed. Ridge regression was used to deal with this multicollinearity issue. Ridge regression penalizes highly correlated terms in the model and so arrives at the range of terms genuinely driving effects. Lambda was automatically selected.

### Land equivalent ratio (LER)

LER was calculated each year of harvest across the whole of the North Field (Fig. 2) as Mead & Willey (1980). Areas of the arable crop and tree understory were measured from Google Maps, accessed 15/07/2024. Crops grown in agroforestry and monoculture assumed all planting and management activities as described above in the *Arable rotation* section. A walnut monoculture model was produced with trees planted in a 3-m-spaced grid with the same unmanaged grass understory as tree line understories. Tree yield was measured as stem plus branch carbon. Alternative measures such as biomass could have been used but they are a simple function of carbon content, and this would make no difference to LER values. Hi-sAFe is not yet parameterized to output realistic values of nut yield.

A full, detailed mechanistic analysis of model output (including ridge regression, LER, and graphical analysis of climatic and crop phenological correlates of crop yield—see results) was only attempted for RCP8.5 output, but core findings (year on year wheat and pea yields) were plotted, analyzed statistically, and compared at both RCP8.5 and RCP4.5. All calculations and statistics undertaken in this study can be examined in the “Summary” and “RCP4pt5” sheets of the “Analysis.xlsx” MS Excel workbook submitted as supplementary material with this article.

## Results

### Model validation

Modelled above-ground tree growth trajectories are shown in Figure SI1 with means and confidence intervals for trees measured in 2023 shown against RCP8.5 modelled data. Model trajectories for 5 different above-ground tree dimensions passed through the 95% confidence intervals for 17 real trees measured in 2023, indicating that aboveground tree characteristics were realistically modelled by Hi-sAFe.

**Fig. 3:**
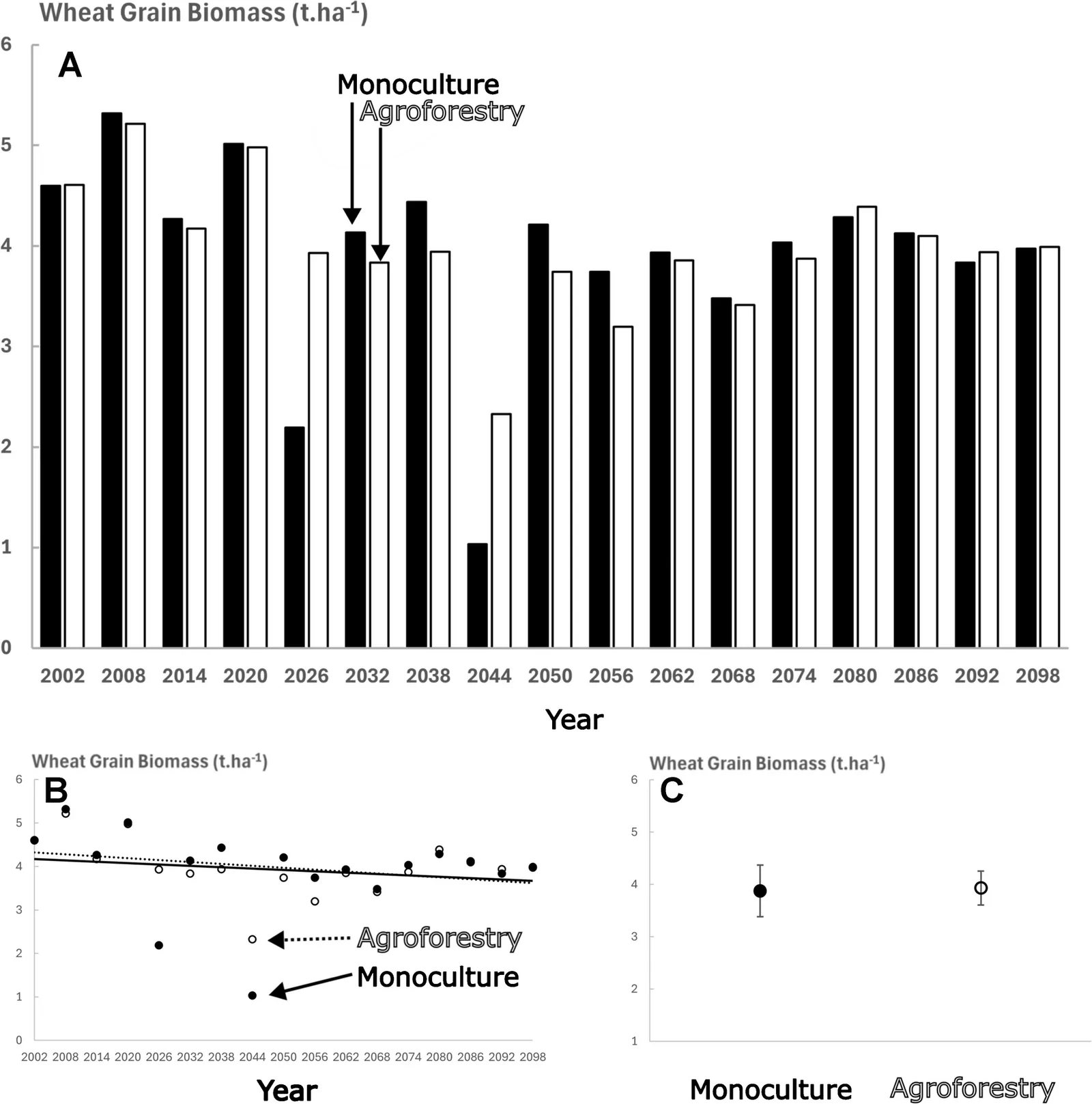
Simulated wheat yield by year under RCP8.5 in agroforestry and monoculture at Wakelyns in Eastern England. In part **B**, linear regressions have been fitted to the data shown in Part **A**. There is no significant downward trend in either line and there is no significant difference in slopes between lines: lm(formula = Yield ~ Year * Group) yielded “Year,” *β* = −0.00522, SE = 0.00700, t(30) = −0.745, *p* = 0.462; “Year*Group,” *β* = −0.00217, SE = 0.00990, t(30) = −0.219, *p* = 0.828. In part **C** variation around the mean was not significantly different between monoculture (black dot) and agroforestry (white dot): Levene’s test for homogeneity of variance, F(1, 32) = 0.516, *p* = 0.478. Bars shown are ± 95% CIs.

While there is little long-term wheat yield data available at Wakelyns, Smith and Westaway (2020) indicate a yield of winter wheat of around 3.2 t/ha in 2009 in tree-lined alleys at the site. The average Hi-sAFe RCP8.5 yield between 2001 and 2100 was 3.93±0.322 t/ha (mean±95%CIs, *n*=17, Fig. 3); somewhat higher, but this could be explained using a conventional commercial wheat variety in the model rather than the mixed genotype YQ wheat grown at Wakelyns. Pea yield predicted by the model was 1.73±0.634 (mean±95%CIs, *n*=16, Fig. 5). Data on organic pea yield is scarce but Caldbeck and Sumption (2016) report organic pea yields across Europe to lie between 1 and 4.5 t/ha.

### Wheat yield under RCP8.5

The principal impact of agroforestry on winter wheat yield at Wakelyns under the RCP8.5 scenario was to protect wheat during occasional years (2026 and 2044 in simulations) of exceptionally low yield in monoculture, i.e., “bad years.” In 2026, yield in monoculture was around 2t/ha but about 4t/ha in agroforestry. In 2044, yield in monoculture was about 1t/ha but nearer 2.5t/ha in agroforestry (Fig. 3). Other qualitative effects were more subtle. Before 2080, agroforestry generally resulted in a very small yield penalty of around 4.5%, excluding the anomalous years of 2026 and 2044. However, from 2080 onwards, there is an indication that yield is either similar or greater in agroforestry than monoculture, although absolute effects are small. This suggests that as climatic extremes intensify, agroforestry at Wakelyns may shift from something that generally restrains wheat yield to something promoting it.

There appears to be a downward trend in wheat yield with year in both agroforestry and monoculture (Fig. 3A and B) but this is not statistically significant and there is no significant difference in the slope of yield declines between agroforestry and monoculture (statistics reflecting these statements are shown in the legend of Fig. 3). There is no significant difference in variation of yield across years with respect to agroforestry and monoculture; i.e., yield is overall no more stable across years in agroforestry than it is in monoculture (Fig. 3C and see legend for statistics).

The largest AgType*Year coefficients in the ridge regression were all associated with Period 3 (March 15–May 31), and each of these three-way terms included a complex interaction of climatic variables (Figure SI2). Simply put, this indicates that year to year variation in yield between agroforestry and monoculture appears to be principally determined in Period 3 and is influenced by complex interactions of all environmental variables considered during this period.

**Fig. 4:**
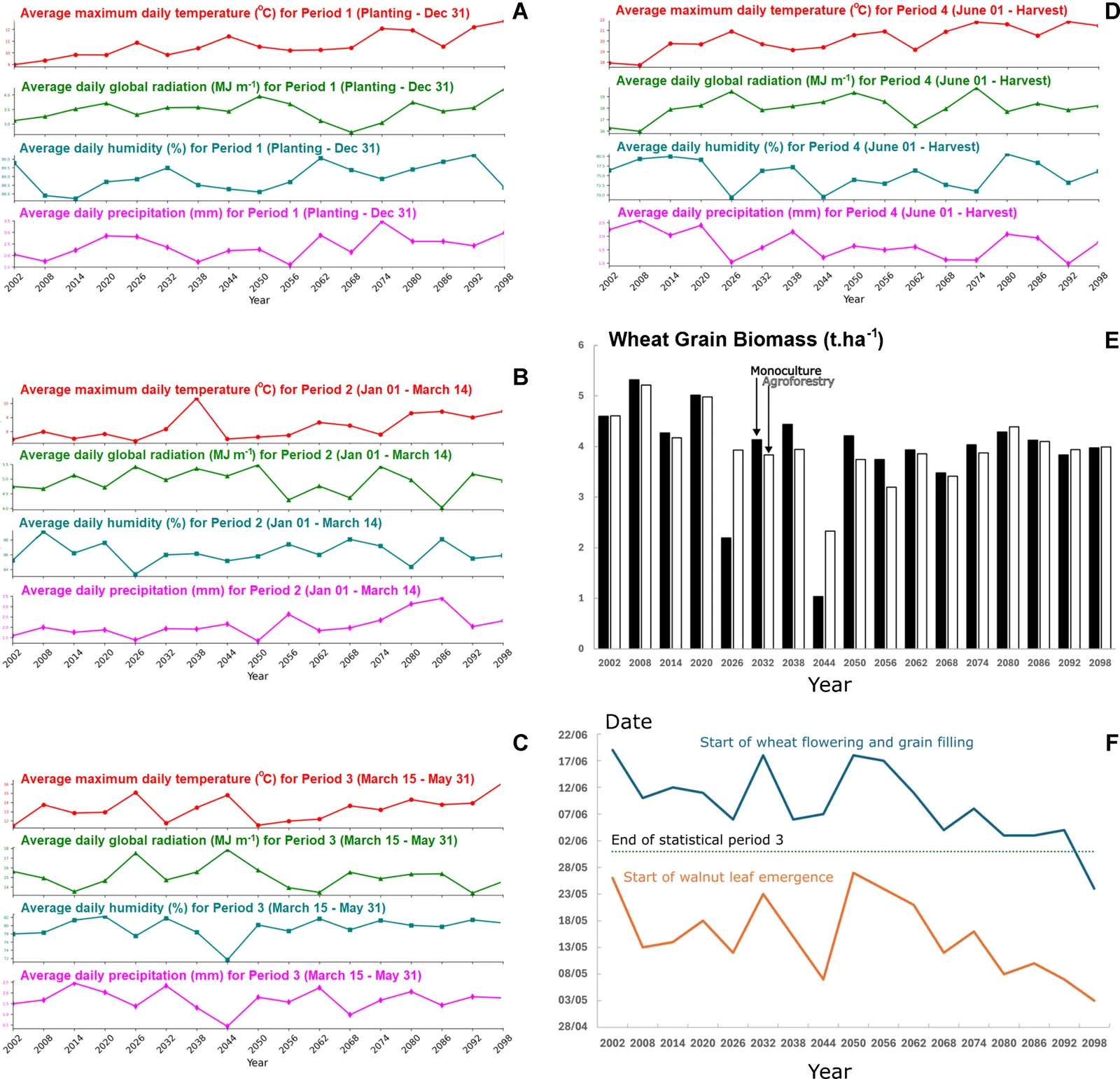
**A**–**D** Average daily values of environmental variables into Hi-sAFe during four periods of the winter wheat growth season at Wakelyns. Part **E** is Part **A** of Fig. 3 reproduced to allow readers to compare yield with climate phenomena (**A**–**D**). Part **F** shows crop and tree phenological events within and after the statistically significant Period 3.

Mean values of environmental variables were plotted in each period along with wheat yield values in Fig. 4A–D. The major environmental anomalies in Period 3 are hot, sunny conditions of low humidity and low precipitation in years 2026 and 2044, when monoculture yield collapses but agroforestry yields remain 79% higher (2026) and 125% higher (2044) (Fig. 4C). In summary, it appeared that the protective effect of agroforestry against a complex combination of hot, sunny, and dry weather conditions between March 15 and May 31 in years 2026 and 2044 of simulations was a significant reason why yield was much higher in agroforestry than monoculture in these years.

This finding is surprising given that March 15–May 31 is usually associated with relatively mild springtime conditions in the UK. To establish what was occurring physiologically in the crop and trees during this period, the major phenological events in crop and walnut trees were plotted (Fig. 4E). The end of Period 3 is a few weeks after first leaf emergence in walnut and a few weeks before the onset flowering and grain filling in wheat. Assuming walnut trees require leaves to exert their protective effects on wheat, this suggests that in 2026 and 2044, years for which the simulated yield was very poor for monoculture, there was a short period of a few weeks immediately after the emergence of walnut leaves when the agroforestry trees protected the physiologically sensitive process of flowering and grain filling in wheat from dry, hot weather and so ensured reasonable yield in the agroforestry system.

### Pea RCP8.5

**Fig. 5:**
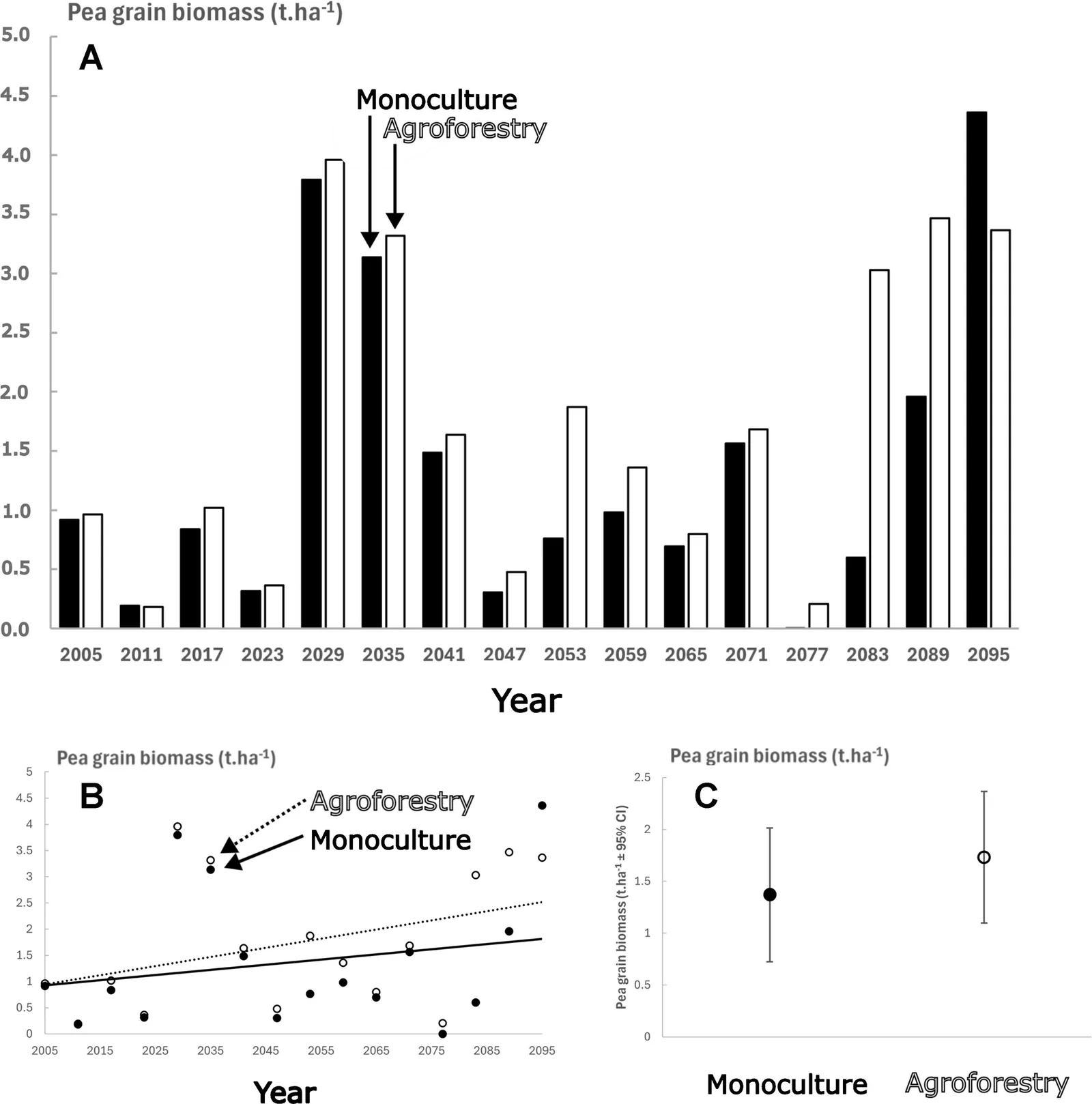
Simulated pea yield by year under RCP8.5 in agroforestry and monoculture at Wakelyns in Eastern England. In Part **B**, linear regressions have been fitted to the data in Part **A**. There is no significant upward trend in either line, the dashed agroforestry line is not significantly higher than the dark monoculture line, and there is no difference in the slope of lines: lm (formula = yield ~ Year * Group (agroforestry vs. monoculture)) yielded: “Year,” *β* = 0.00987, SE = 0.0116, t(28) = 0.851, *p* = 0.402; “Group,” *β* = −15.27, SE = 33.64, t(28) = −0.454, *p* = 0.653; “Year*Group,” *β* = 0.00763, SE = 0.0164, t(28) = 0.465, *p* = 0.646. In Part **C** variation around the mean was not significantly different between monoculture (black dot) and agroforestry (white dot): Levene’s test for homogeneity of variance, F(1, 30) = 0.232, *p* = 0.633. Bars shown are ± 95% CIs.

Qualitatively, Hi-sAFe predicts that pea already benefits from agroforestry at Wakelyns under current conditions (Fig. 5). Across the full period from 2005 to 2071, agroforestry provided an average 26.8% yield advantage for pea relative to monoculture. Generally, this benefit becomes more pronounced through time under RCP8.5. However, variation in yield from year to year and with respect to agroforestry vs. monoculture varied wildly within the 100 years analyzed (pea yield is known to vary pronouncedly through time (Gudko et al. 2024)) and perhaps due to this large variation, the impact of agroforestry vs. monoculture on yield was not significantly different statistically (“Group” term in legend to Fig. 5). There was no significant upward trend in yield with respect to year (“Year” term in legend of Fig. 5). There was no significant difference in the magnitude of the slight upwards slope of yield with time in agroforestry or monoculture (“Year*Group term in Fig. 5). Similarly, there was no significant difference in variation around the mean with respect to agroforestry vs. monoculture (Fig. 5C): agroforestry does not make yield more stable across years. Again, there was an indication that agroforestry protects pea yield from complete collapse in several years, notably in 2053, where agroforestry yield was more than 145% higher than monoculture, in 2077, where agroforestry yield was many orders of magnitude higher due to near-zero monoculture yield, in 2083, where agroforestry yield was 405% higher, and in 2089, where agroforestry yield was 77% higher. Conversely, there was one anomalous year (2095) in which monoculture yield was 23% higher than agroforestry.

Interest centered on identifying the factors responsible for the considerable year-to-year variation in the relative impact of agroforestry vs. monoculture. For example, in 2071, pea yield is quite similar in agroforestry and monoculture (8% higher in agroforestry), but in 2083, it is dramatically greater in agroforestry (405% higher), and in 2095, it is dramatically greater in monoculture (23% higher). Again, ridge regression was used and analysis of the largest AgType*Year coefficients and within-year time period and environmental variables associated with this (Figure SI3). The second largest coefficient in the model was an AgType*Year interaction associated with Period 2 and a complex interaction of environmental variables (RelHumid*MaxDailyTemp*GlbRadiation). Incidentally, the largest term in the ridge regression model described year to year variation in yield and this was also strongly influenced by Period 2 and the same complex interaction of environmental variables (Figure SI3).

**Fig. 6:**
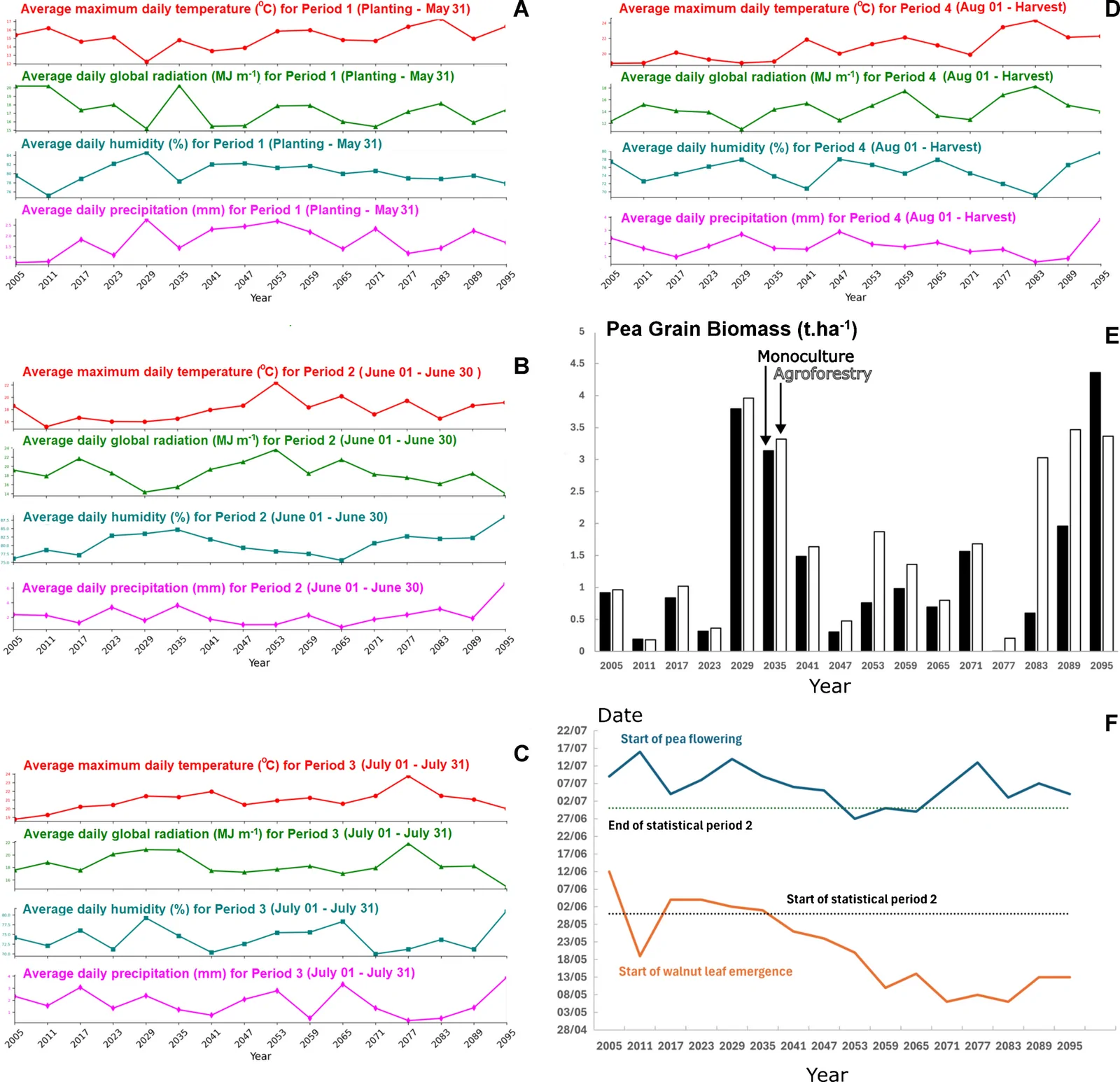
**A**–**D** Average daily values of environmental variables into Hi-sAFe during four periods of the pea growth season at Wakelyns. Part **E** is Part **A** of Fig. 3 reproduced to allow readers to compare yield with climate phenomena (**A**–**D**). Part **F** shows crop and tree phenological events within and after the statistically significant Period 2.

Figure 6A–D show mean values of environmental variables in each period along with pea yield. It is more difficult to attribute variation in yield with particular environmental events in Period 2 as yield variation between agroforestry and monoculture was spread across a number of years and is not principally located in a small number of anomalous years as it is in wheat. Nevertheless, the very high pea yields in 2095 in monoculture are associated with a Period 2 of unusually high precipitation/humidity and low sunlight.

Regardless of our ability to graphically interpret what is happening environmentally in Period 2 and how this impacts yield in agroforestry and monoculture, our statistical tests indicate that Period 2 is key in the different effects of agroforestry and monoculture on pea yield. We, again, plotted some phenological events around this Period 2 (Fig. 6F). In a similar way to what was observed in wheat, this month-long period 2 (June 01–July 30) starts around spring leaf emergence in walnut and ends around the time of flowering and grain filling.

It appears that the relatively early year period between walnut leaf emergence and the initiation of crop flowering and grain filling is fundamental to how both wheat and pea crops respond to agroforestry vs monoculture in our model of Wakelyns agroforestry.

### Land equivalent ratio (LER) under RCP8.5

The results shown so far compare crop yields on arable strips between the trees with yields on open fields without trees. A comprehensive comparison needs to account for the land that is withdrawn from cropping and dedicated to the tree lines (resulting in lower per hectare crop yields), and for the growth of the trees (the alternative product obtained from the tree lines). The LER allows for an overall evaluation of the system’s performance accounting for both crop yields and tree growth (Mead & Willey 1980). Here the LER was computed from predicted crop yields and tree growth (carbon sequestered). The potential nut production from the trees could not be taken into account, as it is not implemented in the Hi-sAFe model as yet.

**Fig. 7:**
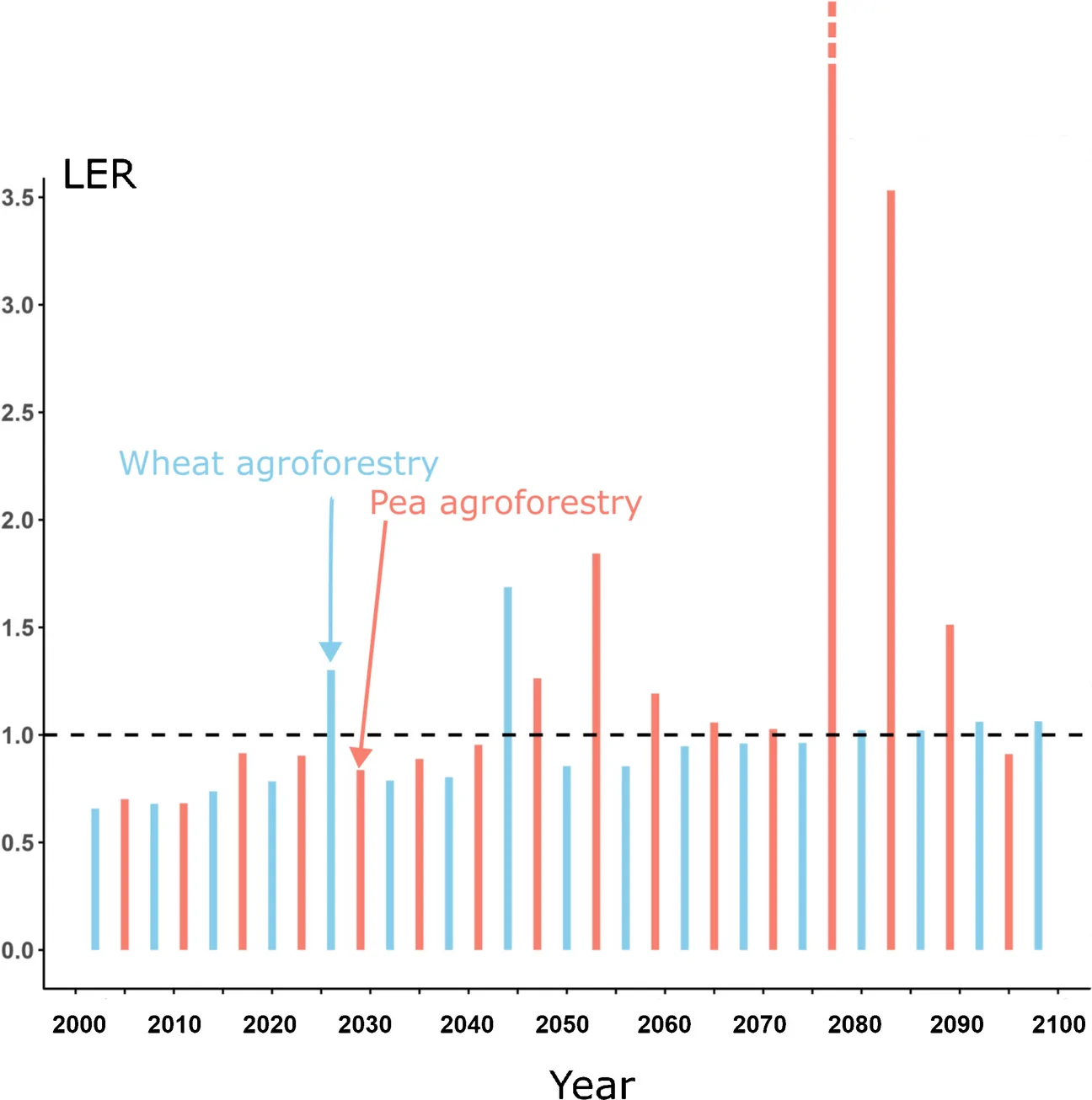
LER of wheat and pea agroforestry in the North Field for RCP8.5. The hugely elevated. 3.5+ LER value in 2077 pea agroforestry is due to the almost complete failure of the crop in monoculture in that year. This near-zero yield value is a denominator in the LER formula, and so LER values are hugely elevated.

Generally speaking, the layout of the North Field at Wakelyns does not appear to be configured in a way that maximizes achievable wheat and tree yield (Fig. 7). LER does not consistently reach 1 until 2080 when, presumably, the growth advantage trees enjoy due to their low-density planting compensates for loss of wheat yield due to competition and wide tree understories. In the exceptional years of 2026 and 2044, agroforestry is a considerably more efficient land use in terms of yield than monoculture.

Growing peas in North Field at Wakelyns appears a more efficient land use than growing wheat. LERs rise above one from around the mid-2040s onwards, presumably due to pea yields being generally higher in AF than MC (Fig. 5). Nevertheless, the fact that it takes 40 years from tree planting to achieve LER > 1 indicates that North Field could be configured more efficiently for yield-related land use.

### RCP4.5

Yield trends in wheat at RCP4.5 were very similar to those at RCP8.5. Across most years, monoculture showed only a small yield advantage over agroforestry (averaging 5.3% from 2002 to 2080, excluding the anomalous 2050 year). However, there were 2 years—2050 and 2086—in which monoculture yield fell sharply and agroforestry yield was markedly higher, by 77% and 20%, respectively (Figure SI4), highlighting the ability of agroforestry to protect wheat yield in “bad years.”

Pea yield trends differed qualitatively from those under RCP8.5 in several respects. First, there were more years of complete yield failure in one or both systems under RCP4.5, occurring in 2011, 2017, 2023, 2035, 2053, 2059, 2071, and 2089 (Figure SI5). Notably, in 2059 and 2071, agroforestry still produced some yield whereas monoculture produced none. Second, in high-yield years—2041, 2065, 2077, and 2095—monoculture yield was typically 15–25% higher than agroforestry, whereas in low- or moderate-yield years—2005, 2029, 2047, and 2083—agroforestry generally outperformed monoculture, by 5–80%, and by much larger margins in years when monoculture approached complete failure. Because agroforestry yield was higher in almost all years with RCP8.5, these results indicate that agroforestry helps pea maintain yield under the most severe climate change scenarios.

## Discussion

### Modelling and empirical studies of crop yield buffering by trees in temperate and tropical regions

In this study, agroforestry buffered wheat yields under both RCP4.5 and RCP8.5, with broadly similar protective effects: in most years, monoculture had only a small average yield advantage, but in occasional bad years with very low monoculture yield, agroforestry maintained substantially higher yields under both scenarios. For pea, however, the protective effect of agroforestry was clearly stronger under RCP8.5 than under RCP4.5: agroforestry out-yielded monoculture in almost all years at RCP8.5, while at RCP4.5, there were more years of complete yield failure in one or both systems and several cases where agroforestry still produced some yield when monoculture failed entirely. Together, this indicates broadly similar buffering for wheat across RCPs, but a pronounced strengthening of pea protection under the more extreme RCP8.5 climate. How do these effects compare with other modelling and empirical investigating the protective effect of agroforestry on arable crops in temperate and tropical regions?

In the Mediterranean, Reyes et al. (2021)—also using the Hi-sAFe model—predicted a protective effect of walnut trees on wheat yields, especially against extreme climate effects, with increased heat stresses due to climate changes reduced by 20–35% in the agroforestry system, which helped to stabilize yields. Giannitsopoulos et al. (2025) calibrated the biophysical EcoYield-SAFE agroforestry model against 1989–2021 field data in Northern Ireland and projected that under RCP 4.5 and 8.5 (2020–2100), elevated CO₂ offsets warming and drought, boosting grass, crop, and timber yields while silvopastoral and silvoarable systems stay productive. Agroforestry and woodland systems preserved or increased soil carbon, whereas pure grassland and arable lands lost 0.43–1.18 t C ha⁻^1^ year⁻^1^. Gomes et al. (2020) simulated Brazilian agroforestry coffee (*Coffea arabica*) systems with 50% shade, demonstrating a reduction in temperatures and maintenance of 75% of the area suitable for coffee production in 2050. Chemura et al. (2021) found protective effects of agroforestry trees for maize yields against climate extremes in Ethiopia. In the regions, where a yield decrease is expected under RCPs 2.5 and 8.5, they predict a yield stabilization of 30%, mostly due to shading resulting in microclimatic temperature stabilization.

Overall, empirical studies in temperate and tropical regions underscore the potential of silvoarable agroforestry to buffer crop yields against extreme weather, contingent upon tree species selection, alley dimensions, and management. At the Ihinger Hof experimental station in Germany, 12 years of yield data for winter cereals and legumes in alley cropping systems revealed significantly lower interannual yield variability during drought years compared to monocultures and tree rows sustained winter wheat and barley yields close to alleys under fluctuating water balance without significant declines (Koch et al. 2025). Swiss studies on winter barley showed that up to 30 % reduction in above-canopy radiation had negligible impact on grain yield and quality when managed appropriately (Vaccaro et al. 2022). In southern Canada, a 27-year-old tree–soybean (*Glycine max*) intercropping system maintained approximately 40% smaller yield reductions under full-season water deficits, attributable to enhanced biological N₂-fixation and moderated soil moisture stress (Nasielski et al. 2015). In tropical environments, improved *Sesbania sesban* fallows in eastern Zambia increased maize grain per unit water use by ~20% and stabilized yields across dry seasons compared to continuous maize (Phiri et al. 2003). Semi-arid Kenyan trials showed that *Grevillea robusta*–based agroforestry systems reduced daytime soil evaporation by ~20% and moderated air and soil temperatures, resulting in more consistent maize yields during rainfall deficits (Ong et al. 2000; Lott et al. 2009). In sub-humid Rwanda, mature *Alnus acuminata* litter inputs offset shade-induced stress during wetter seasons, enhancing maize nutrient uptake without exacerbating water limitations (Ndoli et al. 2017).

While one might expect buffering effects of agroforestry to be more pronounced in the tropics, such a trend is not immediately obvious from the primary literature. A global meta-analysis of 125 studies reported that agroforestry increased soil water content by an average of 40% in temperate systems and 81% in tropical systems, suggesting stronger soil-moisture buffering in the tropics, but yield stability was not considered directly (Ngaba et al. 2024). This issue of the relative buffering strength of agroforestry in temperate and tropical regions is an interesting topic with practical implications for agriculture and deserves a dedicated meta-analysis.

### Issues with model-generated climate data

While considered one of the best-performing RCMs (Jacob et al. 2014; Vautard et al. 2021), RACMO does embody some inherent biases. It uses an ~12-km grid and so, like all RCMs, must rely on simplified parametrizations to represent sub-grid scale processes (e.g., convection, evaporation) that prevent the explicit representation of, for instance, localized heavy rainfall events and intense storms. RCMs often underestimate heavy rainfall when the air is relatively dry, and RACMO in particular shows large spread between different model versions (Lenderink et al. 2025). It also tends to overstate the size of rare downpours by about 15–45% and “flatten” the curve that describes how unlikely very extreme storms are, so a non-stationary peaks-over-threshold correction must be applied to its output (Roth et al. 2014). Finally, its estimate for a given extreme event (like the 50-year rainfall) can jump by 15% versus 45% simply depending on whether it’s driven by ECHAM5 or MIROC global data—showing that the choice of boundary conditions adds substantial uncertainty (Roth et al. 2014). Bias correction using quantile mapping was attempted on the current climate date set but unfortunately only a small number of years of detailed climate data at Wakelyns were available and bias corrections only improved model predictions for wind speed. On the other hand, the impact of climate data biases, especially in climate extremes, may be less pronounced in Hi-sAFe than in some other agronomic models as Hi-sAFe does not, as yet, encode plant mortality, which one might expect to be a major consequence of climate extremes. In Hi-sAFe, plant physiological processes simply slow down or stop during extreme conditions so it might be expected that inaccuracies in reflecting climate extremes are reflected in quantitatively rather than qualitatively different tree and crop growth and yield dynamics.

### Phenological mechanisms underlying buffering effects of agroforestry and their practical implications

It was unexpected that spring-early summer climatic events (around the time of walnut leaf emergence followed by the start of crop flowering) in winter wheat and pea were key determinants of agroforestry vs monoculture crop yield in both these species given that this does not cover the hottest period of the year. This is, of course, a somewhat naive and anthropomorphic response as there is no reason why the phenological sensitivity of plants and humans should be similar, and it is clear from the literature that abnormally high heat, low humidity, or cold during flowering and grain filling are among the key climatic determinants of yield in both these crop species (Saini et al. 1983; Pavek 2012; Karkanis et al. 2016; Hassan et al. 2021; Lamichaney et al. 2021; Zhao et al. 2022; Jiang et al. 2025). In the case of wheat, there was clear evidence that key yield-determining events during this period were driven by high heat and low water–related climate characteristics. This was less obvious in pea but the same phenological period was indicated as important in pea, and comparison of yields at RCP4.5 and RCP8.5 indicates that pea benefits from the presence of trees in the hotter, dryer climate scenario.

How does agroforestry protect the crop during this sensitive period? The most likely mechanism is through the provision of shade and microclimatic modification from the newly emerged walnut leaves. Another possibility, however, is the onset of hydraulic redistribution in the trees due to transpiration in the new foliage. Hydraulic redistribution is defined as, “…the passive movement of water from moist to dry soil through plant roots, including the lifting of water from the deeper to shallower soil layers (hydraulic lift), the movement of shallow to deep soil layers (downward hydraulic redistribution), and lateral transportation.” (Saengwilai et al. 2022). However, such mechanisms are not as yet encoded in Hi-sAFe (Dupraz et al. 2019). Assuming shade is responsible, this indicates that farmers may have some additional points to consider when selecting a tree species for silvoarable agroforestry. Most arable growers configuring a new agroforestry system will be aware that there needs to be a good gross phenological match between crop and trees. For example, evergreen trees should not be used in a field commonly growing winter wheat (as they will shade the crop year-round). However, these new findings suggest some new, early season considerations. Agroforestry trees should be selected to show leaf emergence just before arable crop flowering but presumably not much before to minimize the effects of shade in periods when the tree is “not needed.” Walnut in England is an excellent species in this regard as its leaves emerge late, near the end of May, and not long before flowering begins in the arable crop. White poplar (*Populus alba*) is a common tree in agricultural areas in England but puts out leaves in March or April and so is shading arable crops well before it is “needed” for stress prevention during flowering and grain filling. Assuming effects demonstrated in our model and analysis represent tree and crop behavior in the field, these are additional considerations that the new silvoarable farmer should consider.

### Insights from LER time series

Studies of temperate silvoarable agroforestry commonly obtain LERs of well over one (Morhart et al. 2014; Seserman et al. 2019; Lehmann et al. 2020; Amassaghrou et al. 2023) indicating that agroforestry is a more efficient land use than growing the same trees and crops in monoculture. However, these studies are typically done on mature systems and few empirical studies have the ability to measure LER in time series as in this study and that of Giannitsopoulos et al. (2025). The current study indicates that it would take 80 and 40 years at RCP8.5, respectively, for wheat and pea agroforestry to reach LER>1 and Giannitsopoulos et al. (2025) calculate that silvoarable agroforestry at their Northern Irish site never reaches >1 at RCP8.5 unless tree understories are considered productive. So new silvoarable practitioners in temperate regions are likely to have to wait some time for their system to maximize productivity. This said, it should be appreciated that Wakelyns was not originally conformed with maximum productivity in mind. The site was established in the 1990 s by Professor Martin Wolfe to demonstrate the role of biodiversity in agricultural resilience. Actions that would increase yield productivity include lowering the width of tree understories and planting trees closer together. The current co-owner of the site, Martin Wolfe’s son David who manages the site with his wife Amanda, is taking steps to improve productivity of areas such as North Field, through activities such as planting grape vines in the spaces between trees, so making the tree understory more agriculturally productive. It is likely that with these recent measures incorporated into calculations, LER would be higher than indicated in this paper and the wait to LER > 1 would be much shorter.

## Conclusions

By modelling long-term silvoarable agroforestry performance under contrasting climate-change scenarios to assess yield stability, underlying microclimatic and phenological mechanisms, and land-use efficiency, this study demonstrates for the first time that silvoarable agroforestry can act as a climate shock absorber by preventing catastrophic yield failures during acute stress years. The modelling shows that the magnitude of this buffering effect differs between crops and climate scenarios. For wheat, agroforestry provided broadly similar protection under RCP4.5 and RCP8.5: monoculture yielded slightly more in most years, but in the occasional bad years with very low monoculture yield, agroforestry consistently maintained substantially higher yields. This indicates that agroforestry stabilizes wheat production primarily by reducing downside risk rather than by increasing average-year yield stability. For pea, the protective effect was more pronounced under RCP8.5, where agroforestry outperformed monoculture in almost all years and markedly reduced the severity of yield losses. This shows that agroforestry becomes particularly valuable for maintaining pea yields under the most severe climate-change scenario.

The study also identifies underlying microclimatic and phenological mechanisms that underpin these protective effects. For both wheat and pea, yield buffering occurred during a narrow early-season window corresponding to crop flowering and grain filling, when crops are most sensitive to heat and evaporative stress. Across crops and scenarios, the onset of tree leaf emergence coincided with this vulnerable reproductive period, reducing climatic stress relative to monoculture and thereby limiting the severity of yield losses. Although the strength of protection varied between wheat and pea, the mechanistic basis—tree-induced microclimatic modification aligned with crop phenology—appears consistent across both crops.

In relation to overall system productivity, the land equivalent ratio (LER) showed a substantial initial lag, with values consistently exceeding 1 only after approximately 40–80 years. This lag reflects the original design and management of the Wakelyns system rather than an inherent limitation of agroforestry. The modelling suggests that modified spatial configurations, alternative species choices, or more adaptive tree-management strategies could accelerate the time required to achieve combined productivity gains.

These findings also provide practical guidance for farmers seeking to enhance the climate resilience of arable systems. Because protective effects arise specifically during crop flowering and grain filling, agroforestry systems are likely to perform best when tree leaf emergence overlaps with this early-season sensitivity window. Leaf burst that occurs too early may impose unnecessary shading, while leaf burst that occurs too late will miss the period when protection is most effective. Selecting tree species or cultivars—and managing them accordingly—to align leaf emergence with crop reproductive timing offers a pragmatic, evidence-based strategy for strengthening crop resilience as climatic extremes intensify.

## Data Availability

Supplementary Information MS Word file is available: SI.doc @ https://zenodo.org/records/17843966 or DOI 10.5281/zenodo.17843965. All model parameter files for all scenarios run are available as supplementary material: Hi_sAFe_parameter_files.zip @ https://zenodo.org/records/17843915 or DOI 10.5281/zenodo.17843914. Supplementary material showing all analysis of simulation outputs undertaken is available: Analysis.xlms @ https://zenodo.org/records/17844007 or DOI 10.5281/zenodo.17844006.
